# EEG Microstates During Multisensory Stimulation: Assessing the Severity of Disorders of Consciousness and Distinguishing the Minimally Conscious State

**DOI:** 10.3390/brainsci15121306

**Published:** 2025-12-04

**Authors:** Tao Min, Fangfang Sun, Jiaxue Tong, Zixuan Chen, Yong Yang, Shuai Han

**Affiliations:** 1 School of Automation, Hangzhou Dianzi University, Hangzhou 310018, Chinayyang@hdu.edu.cn (Y.Y.); 2 Department of Neurosurgery, Beijing Tiantan Hospital, Capital Medical University, Beijing 100070, China

**Keywords:** DOC, microstate of EEG, multisensory stimulation, global template

## Abstract

**Background:** The clinical assessment of Disorders of Consciousness (DOC) has long been constrained by the subjectivity of behavioral scales and the low-temporal resolution of neuroimaging techniques. There is an urgent need for objective, high-temporal-resolution biomarkers to improve the accuracy of DOC severity evaluation and sub-state differentiation. This study aims to develop a resting-state/task-state dual-modality EEG microstate analysis method. By integrating a multisensory stimulation paradigm with a resting-state global template, we seek to verify the validity and clinical utility of this method in quantitatively assessing the severity of DOC sub-states, namely Minimally Conscious State-positive (MCS+), Minimally Conscious State-negative (MCS−), and Vegetative State (VS). **Methods:** A total of 27 subjects were enrolled, including 9 healthy controls (HC), 6 MCS+ patients, 6 MCS− patients, and 6 VS patients. A multisensory stimulation paradigm (visual, olfactory, and combined visual–olfactory) was applied, and EEG microstates were extracted using a revised K-means clustering algorithm. Key microstate parameters (duration, global field power, and coverage) were quantified for systematic analysis. **Results:** During the resting state, the HC group exhibited a significantly posterior parietal-dominant distribution of Microstate D, while this parameter showed a gradient attenuation pattern corresponding to the severity of consciousness impairment in the DOC group (*p* < 0.05). During the task state, the HC group showed a significant multisensory effect under combined visual–olfactory stimulation; within the DOC group, MCS+ patients demonstrated stronger task-related responses compared to MCS− and VS patients. **Conclusions:** The gradient attenuation of resting-state Microstate D parameters reflects the severity of DOC, and task-specific responses to multisensory stimulation serve as a potential biomarker for distinguishing MCS+ patients. This dual-modality EEG microstate analysis method provides an objective, high-temporal-resolution basis for the precise clinical evaluation of neurological function in DOC patients.

## 1. Introduction

The accurate evaluation of Disorders of Consciousness (DOC) is still a major challenge within the field of neuroscience. Current clinical evaluations predominantly rely on behavioral scales, which present inherent limitations. The Glasgow Coma Scale (GCS), for instance, neglects assessment of brainstem function [1] and is susceptible to subjective interpretation when evaluating verbal responses in intubated patients [2], potentially resulting in systematic underestimation of consciousness levels. The Coma Recovery Scale-Revised (CRS-R), although regarded as the “gold standard,” [3] also suffers from constrained diagnostic efficacy due to the heterogeneity of behavioral responses, with misdiagnosis rates of approximately 40% between patients in Vegetative State (VS) and those in a Minimally Conscious State (MCS) [4,5]. Furthermore, CRS-R is often incapable of detecting covert consciousness [6]. This diagnostic dilemma becomes even more pronounced in specific populations. In pediatric patients, developmental stage-dependent differences in motor-cognitive coupling may lead to false-negative judgments [7]. Neurodegenerative conditions such as Parkinson’s disease often conceal residual conscious activity in the elderly [7,8]. For patients in prolonged comatose states, elevated sensory stimulation thresholds and difficulties in recognizing micro-expressions present dual challenges to assessment [7]. Adding to the complexity, the state of consciousness exhibits dynamic fluctuations. Approximately 18% of VS patients exhibited MCS-like features across different time-windows [9]. Environmental confounders such as sedative administration and metabolic disturbances further compromise the reliability of behavioral assessments [4,10]. The limitations of behavioral scale assessments not only contribute to uncertainty in diagnosis but also lead to ethical challenges in medical decision making [5]. For example, misunderstandings of terms such as “vegetative state” among family members may result in treatment delays or the misallocation of medical resources [7,11].

Researchers have increasingly turned to developing objective biomarkers using neuroimaging techniques to address the inherent limitations of behavioral assessments. Resting-state functional magnetic resonance imaging (fMRI) studies [12] have demonstrated that the strength of functional connectivity between the posterior parietal cortex and the prefrontal cortex decreases in a stepwise manner with declining levels of consciousness in DOC patients. Specifically, patients in VS exhibit a 38% reduction in connectivity strength compared to those in MCS (*p* < 0.001) [13], with significant correlations observed between these measures and CRS-R scores (r = 0.71) [14,15]. However, conventional neuroimaging techniques such as fMRI are limited by their low-temporal resolution and therefore lack the sensitivity required to observe rapid shifts in consciousness levels [16,17]. Importantly, the rate of false negatives in fMRI can be as high as 29%, with some behaviorally unresponsive patients displaying covert consciousness patterns on fMRI scans [18].

In this context, EEG microstates are transient and stable spatial topographic patterns of scalp EEG activity that act as “functional building blocks” of whole-brain networks. They capture synchronized large-scale neural activity over short time windows and are typically categorized into four canonical classes (A, B, C, and D) in both healthy and clinical populations. Among these classes, Microstate D is specifically linked to the default mode network (DMN), which is a core network for conscious awareness. Key microstate parameters, including duration, coverage, and transition rate, directly reflect the integrity and flexibility of brain networks, rendering them sensitive to pathological changes associated with consciousness disorders. EEG microstates capture the dynamic reorganization of whole-brain networks with millisecond precision, typically ranging from 80 to 120 ms, which allows them to overcome the temporal-resolution limitations of conventional neuroimaging methods. For example, studies have shown that individuals who regained consciousness exhibited a 25% increase in microstate transition entropy, indicating reconstruction of brain network flexibility [19]. Multimodal evidence has further linked electrophysiological activity measured by EEG to metabolic activity within the DMN. Operational synchrony in EEG is positively correlated with FDG-PET metabolic rate (r = 0.78), whereas patients in a Vegetative State show only 30% of the synchrony observed in healthy individuals [18]. The functional integrity of the DMN has been consistently linked to levels of consciousness [19]. Structural evidence supports these findings: diffusion tensor imaging (DTI) studies have reported a 32% reduction in fractional anisotropy (FA) in the cingulum bundle of patients in VS, indicating functional disconnection within the DMN [20]. Importantly, dysfunction in the posterior parietal cortex appears to be consistent across different pathologies. In schizophrenia, it manifests as attenuated frontoparietal connectivity (effect size d = 1.2), whereas in posterior cortical atrophy, it results in a 60% reduction in visual network efficiency [13,21]. In EEG frequency-domain studies, microstate sequences in the alpha band have been shown to precisely characterize the reorganization of neural assemblies involved in visuomotor coordination, with a temporal resolution of approximately 10 ms [22]. Graph-theoretical network analyses have further revealed aberrant topological configurations within the frontoparietal microstate network in schizophrenia [16,23,24]. From a functional network perspective, neuroimaging studies demonstrate that connectivity in the posterior parietal node of the DMN declines quantitatively with decreasing consciousness [25], consistent with EEG microstate analyses showing posterior parietal dominance in microstate distribution [22,26]. Collectively, these findings suggest that posterior parietal-driven EEG microstates, particularly Microstate D, may serve as reliable electrophysiological markers of DMN dynamics and provide insight into the neural basis of consciousness.

Building upon these findings, the present study employed a multisensory stimulation paradigm and present a resting-task dual-modal validation method to systematically investigate the dynamic response characteristics of microstates. Experimental results revealed that, during the resting state, Microstate D exhibited a posterior parietal-dominant topographic distribution, which was most prominent in the HC group and progressively decreased with increasing severity of DOC. In task-state condition, MCS+ patients demonstrated stronger responses under combined visual–olfactory stimulation compared to MCS− and VS patients. These findings suggested that Microstate D analysis overcomes the limitations of traditional analyses confined to a single spatial or temporal dimension. Parameters derived from resting-state Microstate D may serve as objective and quantifiable indicators of the severity of DOC. During task conditions, the distinct responses in MCS+ patients indicate that Microstate D may serve as a differential diagnostic biomarker. Microstate D may serve as a dynamic network marker for DOC, offering a novel basis and direction for precise clinical assessment.

The core research question of the present study is to determine whether dual-modal (resting-state and task-state) Microstate D analysis can serve as an objective electrophysiological biomarker to improve DOC severity assessment and the differential diagnosis of MCS+, MCS−, and VS. This study addresses the current research gaps, including the subjectivity of behavioral scales, the limited temporal resolution of conventional neuroimaging, and the lack of validated electrophysiological indicators for DOC subtyping. It also builds on evidence that the integrity of DMN is associated with consciousness levels and that posterior parietal cortex-related microstates reflect DMN activity. Based on this rationale, the study proposes two hypotheses: Hypothesis 1: Resting-state Microstate D, viewed as a proxy for DMN functional activity, will show altered topographic distribution and changes in key parameters such as coverage and duration in DOC patients compared with healthy controls. These alterations are expected to correlate positively with consciousness level, with more preserved Microstate D features in individuals with higher levels of consciousness. Hypothesis 2: Multisensory stimulation, particularly combined visual and olfactory input, will modulate Microstate D dynamics in the task state. The strength of these modulatory effects is expected to differ across DOC subtypes. Because MCS+ patients retain partial higher-order cognitive functions, they are expected to display more pronounced and distinguishable Microstate D responses than MCS− and VS patients.

## 2. Methods

### 2.1. Participants

A total of 41 individuals with suspected DOC were initially screened at Beijing Tiantan Hospital. Participants were eligible for inclusion if they met the CRS-R-based diagnostic criteria for MCS+, MCS−, or VS, were clinically stable prior to EEG acquisition, and were able to safely undergo the multisensory stimulation paradigm. Exclusion criteria included acute metabolic or infectious disturbances, major psychiatric conditions, structural neuroimaging findings inconsistent with DOC etiology, and excessive EEG artifacts that compromised data reliability. Fourteen individuals were excluded based on these criteria: six due to metabolic or infectious conditions, three due to incompatible neuroimaging findings, and five due to poor EEG signal quality. The final sample comprised 27 participants, consistent with the distribution reported in the main text (HC = 9; MCS+ = 6; MCS− = 6; VS = 6).

The study was sanctioned by the Ethics Committee of Beijing Tiantan Hospital, Capital Medical University, and was performed in accordance with the Declaration of Helsinki (Ethics Approval No. KY2023-175-03). All participants were recruited based on strict inclusion and exclusion criteria to ensure sample homogeneity and the validity of the experimental results. Written informed consent was obtained from all participants following a detailed explanation of the study aims, methods, and associated risks. This study was under the continuous oversight of the Ethics Committee to guarantee strict adherence to international ethical standards for medical research.

### 2.2. Experimental Design

**Figure 1:**
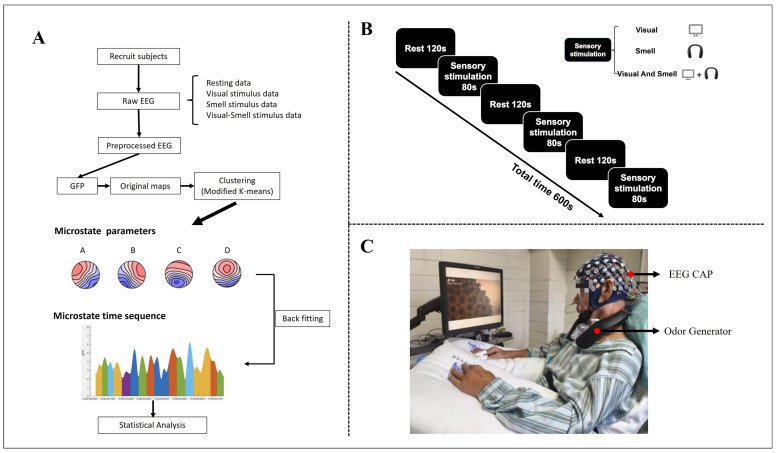
Schematic diagram of experimental procedures (**A**), paradigm (**B**), and data acquisition (**C**).

A multisensory stimulation paradigm was employed in this study, in which each participant completed three types of experimental conditions in sequence: unimodal visual (V), unimodal olfactory (O), and combined visual–olfactory (V-O) stimulation. Each condition consisted of three independent trials, and each trial followed a fixed structure comprising a resting state (120 s), a task state (80 s), and a post-task resting state (120 s) [27,28]. The total duration of each experimental condition was approximately 10 min. The full experimental procedure is illustrated in Figure 1B. E-Prime 3.0 software was employed to oversee stimulation presentation and timing, ensuring temporal precision in the experimental protocol.

Visual stimulation, consisting of silent dynamic video clips, was delivered via a 28-inch display monitor operating at 120 Hz. The visual content included cleaning-related scenes (e.g., hair washing), food-related scenes (e.g., hotpot meat dipping, and durian peeling), and baseline control segments (resting with a blank screen), ensuring standardized visual input across all conditions.

**Figure 2:**
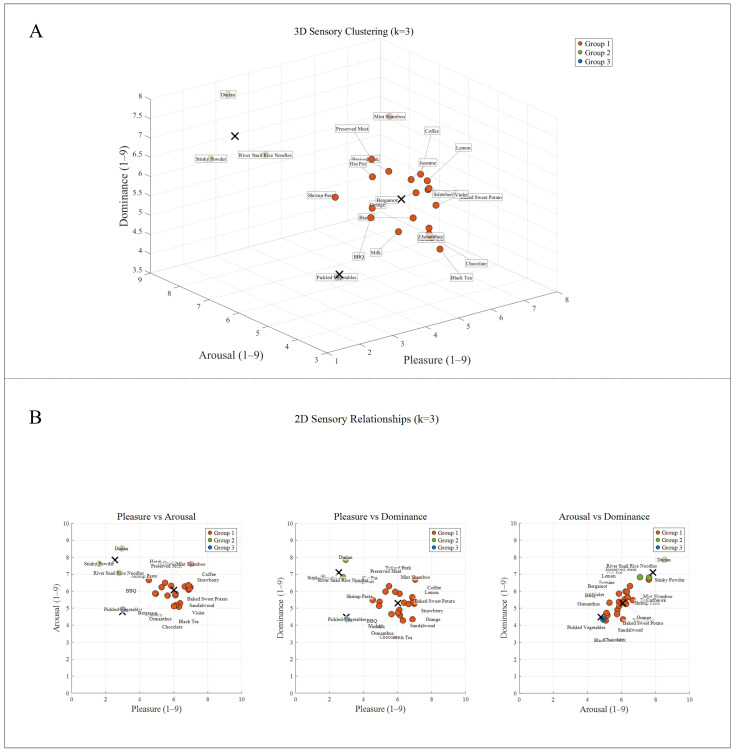
K-means clustering results of 16 odors.

The selection and presentation of olfactory stimuli followed a standardized procedure involving subjective rating, cluster analysis, and calibration of physical delivery parameters. A total of 17 healthy participants were recruited to evaluate 16 odors using the Self-Assessment Manikin (SAM) scale, rating each stimulus on three dimensions: valence (pleasantness, V), arousal (A), and dominance (D). Subsequently, K-means clustering was performed on the SAM ratings to identify three representative odor groups. Figure 2 presents the clustering results: (A) a 3D scatter plot illustrating the spatial distribution of the three groups across the V/A/D dimensions, and (B) 2D relational plots highlighting clustering differences via pairwise dimensional relationships. The three distinct groups were defined as: Group 1 (high-valence low-arousal, e.g., mint shamboo; 7.2 ± 0.8/4.8 ± 0.6/4.5 ± 0.7), Group 2 (low-valence medium-arousal, e.g., durian; 2.3 ± 0.5/5.2 ± 0.5/4.9 ± 0.8), and Group 3 (medium-valence high-arousal, e.g., hotpot; 4.6 ± 0.7/6.8 ± 0.9/5.3 ± 0.6). Finally, an S400 olfactometer (Judian Technology Co., Ltd., Hangzhou, China)delivered the olfactory stimuli at a flow rate of 0.5 L/min, with the stimulation duration synchronized to the 80 s task-state period of the visual condition.

Multimodal stimulation was synchronized using Broadcast Player software (version 1.0), ensuring a maximum onset delay of ≤10 ms between the visual trigger signal and the onset of olfactory delivery. For combined stimulation conditions, semantically congruent visual–olfactory pairings were used (e.g., hotpot video paired with hotpot odor). During unimodal conditions, the non-target sensory modality was completely suppressed to avoid cross-modal interference. The experimental setup is illustrated in Figure 1C.

### 2.3. EEG Processing

The EEG data was collected using a NeuroCloud EEG-3200 system from Yunsen Technologies in Beijing, China, with all main parameters and settings adhering to global electroencephalography standards. The system employed a 32-channel Ag/AgCl electrode cap, and data were recorded with a band-pass filter set between 0.5 and 50 Hz. Electrode impedance was continuously monitored throughout the experiment, and all channels were maintained below 5 kΩ to ensure signal quality. Independent Component Analysis (ICA) [29,30] was applied to remove artifacts associated with ocular and muscular activities. Components correlated with physiological noise were identified and excluded. Raw EEG data were stored offline in EDF+ format for further analysis.

### 2.4. Microstate Analysis

In this study, EEG microstate analysis was implemented using the Microstate EEGLAB Toolbox [31], a plugin for the EEGLAB environment [32], and the complete processing workflow is illustrated in Figure 1A. Microstate prototypes were identified by detecting peaks in the global field power (GFP) [32]. GFP reflects the spatial standard deviation of the scalp electric field at each time point; thus, GFP peaks correspond to moments when the topographic configuration is maximally stable and most representative of underlying neural activity. Using GFP peaks ensures that microstate templates are derived from robust and noise-resistant topographies. Microstates are short-lived (approximately 60–120 ms), quasi-stable scalp potential maps that reflect transient activation of large-scale functional brain networks. Therefore, clustering GFP-peak maps enables extraction of the dominant spatial patterns that constitute the canonical four microstate classes (A–D). For each GFP-peak map, Global Map Dissimilarity (GMD) was computed, and an improved k-means clustering algorithm [32] was used to group maps into four microstate classes. The clustering was performed without additional normalization, using up to 500 iterations, a convergence threshold of 1 × 10^−5^, and 10 random initializations to avoid local minima. The optimal number of microstate classes was determined using the cross-validation (CV) criterion [32,33], and the four-class solution was selected because it yielded the lowest CV value and is widely supported in the prior microstate literature.

After generating the microstate templates, continuous EEG data were backfitted by assigning each time point to the microstate map with the highest spatial correlation. A temporal smoothing procedure was applied, reassigning segments that did not meet the minimum duration threshold to the most likely neighboring class to ensure physiological plausibility and to reduce noise-driven rapid switching.

To ensure comparability across individuals and consciousness levels, a unified global template strategy was used. A resting-state global template was constructed by pooling resting EEG data from all participants, while a task-state global template was constructed by integrating EEG recorded during visual, olfactory, and combined visual–olfactory stimulation. This approach ensures that the templates capture shared spatial patterns across different sensory conditions and consciousness levels [31,34]. All microstate segmentation and parameter extraction were performed based on these global templates.

From the resulting microstate sequences, standard temporal parameters including duration, occurrence rate, and coverage were computed to quantify the dynamic properties of large-scale neural activity.

### 2.5. Statistical Analysis

IBM SPSS Statistics version 27 was used to conduct statistical analyses. Separate analyses were performed for resting-state and task-state data, employing both parametric and nonparametric methods. For the resting-state analysis, the primary objective was to compare microstate parameters across four groups. The noise-resistant topographies Shapiro–Wilk test and Levene test were used to assess normality and homogeneity of variance [35,36], respectively (*p* > 0.05). When both assumptions were fulfilled, one-way analysis of variance (ANOVA) was employed to compare group means, with effect sizes reported as partial eta squared (ηp^2^). If the ANOVA showed a significant main effect (*p* < 0.05), Bonferroni correction was used in conducting pairwise comparisons to control for multiple testing [37,38], and Cohen’s d was calculated to indicate the magnitude of group differences. When the data failed to meet assumptions of normality or homogeneity of variance, the Kruskal–Wallis H test was applied, with effect size measured by epsilon squared (ε^2^),and this was followed by Dunn–Bonferroni post hoc comparisons [39]. Task-state data were analyzed using linear mixed-effects models (LMM) [40] with stimulus type (V, O, V-O) and group (HC, MCS+, MCS−, VS) defined as fixed effects, and participant intercepts as random effects. Restricted maximum likelihood (REML) was used to fit the models, and the Satterthwaite approximation was employed to estimate degrees of freedom. In addition, all within-group comparisons across the three stimulation conditions were also evaluated using the same LMM framework by specifying stimulus type as a fixed effect and participant as a random intercept. This replaces the previous use of separate ANOVA or paired *t*-tests and ensures that all task-state inferences properly account for the repeated-measures design. When significant interaction effects were detected (*p* < 0.05), further analyses were conducted to examine within-group (stimulations condition comparisons within the same group) and between-group (group comparisons under the same stimulus condition) differences. Paired *t*-tests were applied to normally distributed data and Wilcoxon signed-rank tests to non-normally distributed data for within-group comparisons [40], with effect sizes reported as Cohen’s d and rank–biserial correlation, while between-group comparisons made use of independent-samples *t*-tests or Mann–Whitney U tests as appropriate, accompanied by corresponding effect size measures (Cohen’s d or rank–biserial correlation). We considered that when the *p*-value was less than 0.05, the research results were statistically significant. For the interpretation of effect sizes, we followed commonly accepted benchmarks. Specifically, small, medium, and large effects for ηp^2^ and ε^2^ were defined as values of 0.01, 0.06, and 0.14. For Cohen’s d, the corresponding thresholds were set at 0.2 for a small effect, 0.5 for a medium effect, and 0.8 for a large effect. Similarly, for rank–biserial correlation, values of 0.1, 0.3, and 0.5 were considered to represent small, medium, and large effects.

## 3. Result

### 3.1. Demographic and Clinical Characteristics

The four groups (HC, MCS+, MCS−, and VS) did not differ significantly in terms of age, sex and education (*p* > 0.05). Detailed demographic and clinical characteristics are presented in Table 1. Baseline CRS-R scores demonstrated that the HC had a mean score of 22.8 ± 0.1 (mean ± standard deviation, SD), followed by the MCS+ with 17.8 ± 1.17, the MCS− with 10.3 ± 1.03, and the VS with 6.2 ± 1.72. ANOVA performed on all 27 subjects indicated a significant difference in CRS-R scores among the groups, F(3, 23) = 337.76, *p* < 0.001, ηp^2^ = 0.97, which indicates that group differences explain 97% of the variance in CRS-R scores, representing an extremely large effect size and reflecting extremely significant differences in consciousness level-related scores among groups.

### 3.2. Microstate Topographic Map

**Figure 3:**
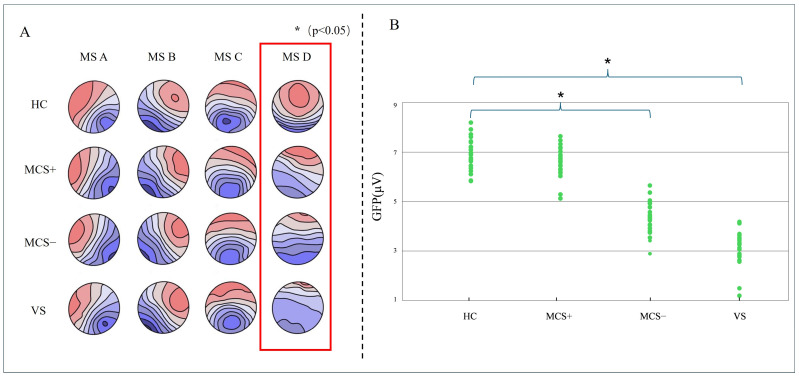
Resting-state microstate topographies across groups (HC, MCS−, and VS patients). (**A**) Group-level microstate topography and GFP peak distribution (**B**).

Using a modified k-means clustering algorithm, the study extracted a global template from resting-state EEG data, yielding four microstate topographies. Temporal analysis of the topographic variance (TANOVA) revealed no significant group differences for microstates A, B, and C (all *p* > 0.05), with spatial configurations consistent with those reported in healthy populations [41]. In contrast, Microstate D, characterized by a posterior parietal dominance, differed significantly between HC and DOC patients (*p* < 0.05). The occurrence of Microstate D decreased progressively with the severity of consciousness impairment. While the HC group showed a stable posterior parietal distribution with a mean occurrence of 23.5 ± 3.2% (mean ± standard deviation, SD), values in DOC patients were reduced as follows: 15.1 ± 2.8% in MCS+, 9.4 ± 2.1% in MCS−, and 4.7 ± 1.9% in VS. ANOVA revealed that these group differences were highly statistically significant and associated with an extremely large effect size (F(3, 23) = 69.54, *p* < 0.001, ηp^2^ = 0.90) which means group differences account for 90% of the variance in the occurrence rate of Microstate D, indicating an extremely large effect size and a strong impact of consciousness impairment severity on the occurrence rate of Microstate D (Figure 3A). Further analysis indicated that the GFP of Microstate D varied substantially across groups, with values presented as mean ± SD: 7.1 ± 0.6 μV in HC, 6.7 ± 0.7 μV in MCS+, 4.4 ± 0.7 μV in MCS−, and 3.1 ± 0.6 μV in VS. ANOVA confirmed a significant overall difference with a very large effect size (F(3, 23) = 58.31, *p* < 0.001, ηp^2^ = 0.89). Pairwise comparisons revealed that while the MCS+ group did not differ significantly from HC (*p* > 0.05), both the MCS− and VS groups showed significant reductions in GFP compared to HC (MCS−: t = 7.99, adjusted *p* < 0.01; VS: t = 12.66, adjusted *p* < 0.01). These findings suggest a marked reduction in posterior parietal neural activity in DOC patients.

### 3.3. Rest State Microstate D Parameter

**Figure 4:**
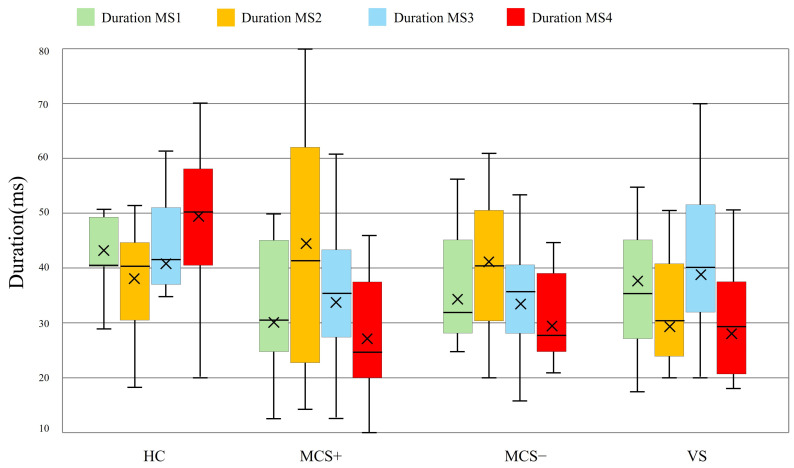
Duration of microstates in DOC patients and HC at rest state. Boxplots depict the distribution of microstate durations for HC, MCS+ and MCS−, and VS groups. Boxplots show 1st quartile, median, and 3rd quartile as well as mean values (large cross).

Back-fitting of resting-state data across all participants revealed notable group differences in the duration of Microstate D, particularly when compared to HC. The duration of Microstate D was 50 ± 20 ms (mean ± standard deviation, SD) in HC, 29 ± 15 ms in MCS+, 30 ± 11 ms in MCS−, and a further reduction to 24 ± 8 ms in VS. These distributions of Microstate D duration are clearly visualized in Figure 4. ANOVA was conducted to assess overall variations in Microstate D duration across the four groups, confirming a statistically significant main effect of group (F(3, 23) = 4.71, *p* < 0.01, ηp^2^ = 0.38), indicating that group differences account for 38% of the variance in Microstate D duration, representing a large effect size (moderate-to-large) and a significant impact of consciousness level on Microstate D duration. Pairwise comparisons further delineated specific group differences: relative to HC, MCS+ participants exhibited significantly shorter Microstate D duration (*p* = 0.016), MCS− participants also showed a significant reduction (*p* = 0.044), and VS participants displayed the most prominent shortening of Microstate D duration compared to HC (*p* = 0.038); notably, no significant group effects were detected for the durations of microstates A, B, or C (*p* > 0.05), indicating that alterations in microstate duration were specific to Microstate D rather than a general microstate abnormality. These findings collectively indicated that the duration of Microstate D gradually shortens as consciousness level declines in DOC. Given the specificity of this alteration to Microstate D and its clear association with consciousness impairment, these results supported the potential of resting-state Microstate D parameters as a novel biomarker for evaluating consciousness status in DOC patients.

### 3.4. Differences in Global Templates Between the Task-State and Resting-State

**Figure 5:**
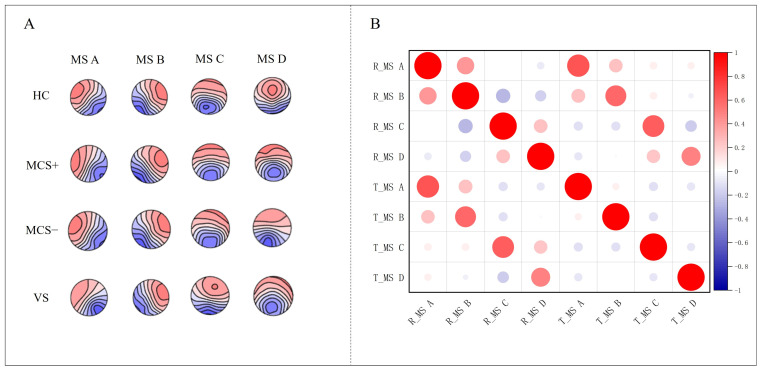
(**A**) shows topographic maps of four microstates (MS A–D) for rest-state and task-state templates. (**B**) presents a heatmap of spatial correlations between the microstate topographies of two templates.

To assess the potential impact of template selection on task-state analyses, a task-state global template was constructed from the combined task-state dataset. TANOVA revealed no significant differences in the spatial distributions of the four microstate classes between the task-state and resting-state templates (*p* > 0.1), as shown in Figure 5A, indicating that task stimulation did not substantially alter the fundamental spatial patterns of EEG microstates. Spatial correlation analysis further demonstrated strong concordance between the corresponding microstate topographies of the two templates (MS A r = 0.82, MS B r = 0.72, MS C r = 0.76, MS D r = 0.71; *p* < 0.001), as illustrated in the heatmap (Figure 5B), supporting the similarity in their spatial topological organization.

**Figure 6:**
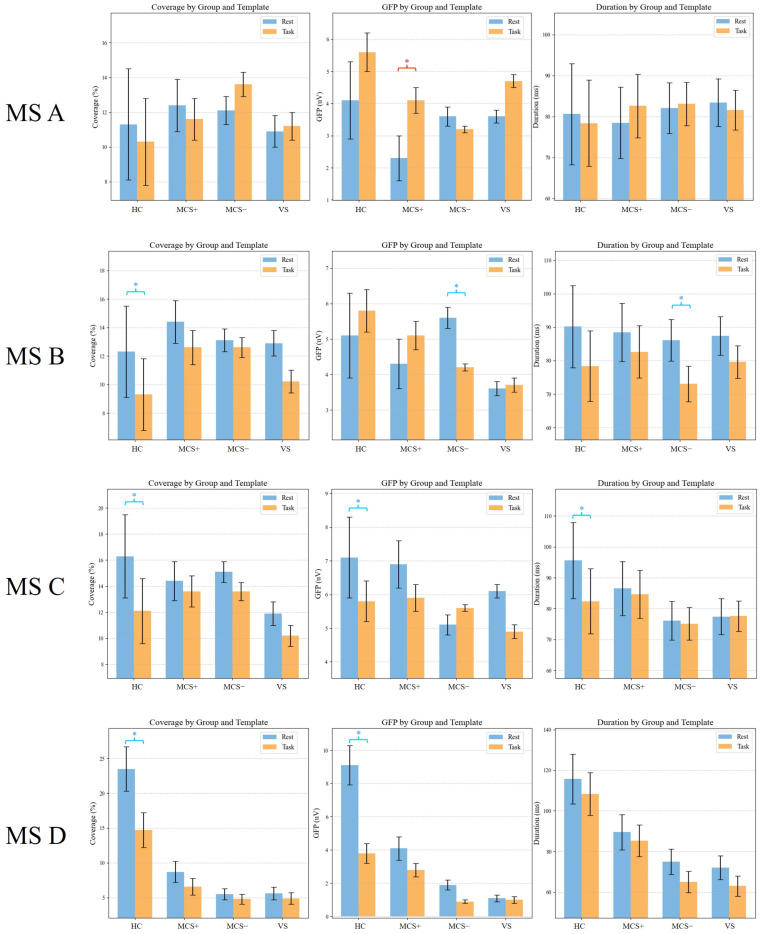
Quantitative analysis of microstate parameters (coverage, GFP, and duration) for MS A–D across groups (HC, MCS+, MCS−, and VS) under resting-state and task-state templates. Red * indicates task > rest (*p* < 0.05); blue * indicates rest > task (*p* < 0.05).

To examine the influence of template type on task-state analyses, we performed back-fitting using visuo-olfactory combined task data against both the task-state and resting-state global templates, followed by quantitative comparisons of microstate parameters. For Microstate A (Table 2), only the MCS+ group showed a significant difference in GFP, with lower values for the resting global template (2.4 ± 0.6 μV) compared to the task global template (4.1 ± 0.4 μV, *p* < 0.05, Cohen’s d = 3.33). Cohen’s d = 3.33 far exceeds the threshold of 0.8 for a large effect size, representing an extremely large effect size and indicating an extremely significant practical difference in the GFP of Microstate A between the two templates in the MCS+ group. For Microstate B (Table 3), the HC group showed significantly higher coverage in the resting global template (12.1 ± 3.4%) than in the task global template (9.6 ± 2.9%, *p* < 0.05, Cohen’s d = 0.79). Cohen’s d = 0.79 is close to the 0.8 threshold for a large effect size, representing a moderate-to-large effect size and indicating a relatively significant practical difference in the coverage of Microstate B between the two templates in the HC group. In the MCS− group, both GFP (resting: 5.6 ± 0.3 μV; task: 4.2 ± 0.1 μV, *p* < 0.05, Cohen’s d = 6.36) (Cohen’s d = 6.36 represents an extremely large effect size, indicating an extremely significant practical difference in the GFP of Microstate B between the two templates in the MCS− group) and duration (resting: 87.1 ± 6.3 ms; task: 74.6 ± 3.9 ms, *p* < 0.05, Cohen’s d = 2.38) (Cohen’s d = 2.38 represents an extremely large effect size, indicating an extremely significant practical difference in the duration of Microstate B between the two templates in the MCS− group) differed significantly. For Microstate C (Table 4), the HC group exhibited significant differences across all three parameters. coverage was higher for the resting global template (16.1 ± 3.2%) than for the task global template (12.1 ± 2.3%, *p* < 0.05, Cohen’s d = 1.44) (Cohen’s d = 1.44 represents an extremely large effect size, indicating an extremely significant practical difference in the coverage of Microstate C between the two templates in the HC group). GFP was also greater in the resting global template (7.1 ± 1.2 μV) compared with the task global template (3.8 ± 1.1 μV, *p* < 0.05, Cohen’s d = 2.87). (Cohen’s d = 2.87 represents an extremely large effect size, indicating an extremely significant practical difference in the GFP of Microstate C between the two templates in the HC group). Similarly, duration was longer in the resting global template (97.6 ± 10.2 ms) than in the task global template (82.3 ± 11.9 ms, *p* < 0.05, Cohen’s d = 1.38). (Cohen’s d = 1.38 represents an extremely large effect size, indicating an extremely significant practical difference in the duration of Microstate C between the two templates in the HC group). For Microstate D (Table 5), significant differences in the HC group were observed for coverage (resting: 23.5 ± 3.2%; task: 14.7 ± 4.1%, *p* < 0.05, Cohen’s d = 2.4) (Cohen’s d = 2.4 represents an extremely large effect size, indicating an extremely significant practical difference in the coverage of Microstate D between the two templates in the HC group) and GFP (resting: 14.7 ± 4.1 μV; task: 3.8 ± 1.1 μV, *p* < 0.05, Cohen’s d = 3.78) (Cohen’s d = 3.78 represents an extremely large effect size, indicating an extremely significant practical difference in the GFP of Microstate D between the two templates in the HC group). These quantitative findings are visually summarized in Figure 6. Overall, the HC group exhibited a greater number of significantly different parameters between the two global templates, including multiple metrics in Microstates B, C, and D, with values generally higher in the resting template. In contrast, DOC patients showed fewer differences, limited to selected parameters in Microstates A and B, accompanied by smaller effect sizes. These findings suggest that healthy individuals have more pronounced EEG features during the resting state, indicating greater stability of the resting template. Conversely, impaired neural function in DOC patients may reduce the capacity to transition between resting and task-related patterns, thereby weakening resting-template stability.

The study further compared goodness-of-fit metrics between different global template (Table 6). It was determined that in the HC group, the task-state data was fitted significantly better by the resting-state global template than by the task-state global template, showing a 42% increase in R squared values (0.78 in contrast to 0.55). In contrast, for the MCS− and VS groups, the standard deviation of residuals for the task-state global template was 6.1 with a standard deviation of 0.9, around 1.3 times higher than the resting-state global template, which was 4.8 with a standard deviation of 0.7. These results indicate that although the resting-state and task-state global template share consistent spatial structures, dynamic disturbances in task-state EEG signals impair global template construction. Due to its greater stability, the resting-state global template is better suited for characterizing brain networks under pathological conditions. Therefore, all analyses of task-state data in this study are based on the resting-state global template.

### 3.5. Dynamic Response of Task-State Microstate D

**Figure 7:**
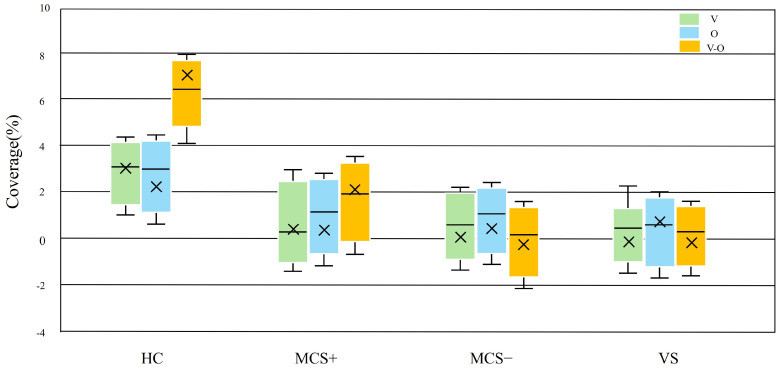
Task-induced changes in Microstate D coverage across groups. Boxplots depict the differences in Microstate D coverage between task (visuo-olfactory combined, visual-only, and olfactory-only stimulation) and resting states.

Given that Microstate D was the only microstate showing a graded difference across DOC severity levels during the resting state, the task-state analysis focused on the dynamic modulation of Microstate D. The linear mixed-effects model revealed a significant interaction between stimulus type and group (F(6, 216) = 4.92, *p* = 0.042), indicating that the effect of multisensory stimulation on Microstate D differed across consciousness levels (Figure 7). In HC group, follow-up LMM contrasts showed that combined visual–olfactory stimulation elicited significantly greater increases in Microstate D coverage compared with unimodal visual or olfactory stimulation. The estimated coverage changes were approximately 3.1 percent for V, 2.7 percent for O, and 7.2 percent for V-O. The overall effect of stimulus type within HC remained significant under the LMM framework (F(2, 24) = 9.81, *p* < 0.001, ηp^2^ = 0.24). Follow-up pairwise LMM contrasts confirmed that V-O stimulation resulted in significantly greater increases than both V (*p* = 0.003) and O (*p* = 0.007). Among DOC patients, only the MCS+ group demonstrated a modest but detectable increase in Microstate D coverage in response to V-O stimulation (2.1 percent), whereas the MCS– and VS groups showed no significant task-related modulation under any stimulation condition (all LMM contrasts *p* > 0.1). These findings indicate that preserved task-evoked flexibility of Microstate D is specific to the MCS+ subgroup.

## 4. Discussion

This study systematically investigated the EEG characteristics of DOC and the diagnostic potential of Microstate D through analysis of resting-state and task-state EEG microstates. These results demonstrated that the posterior parietal-dominant Microstate D exhibited significant features closely associated with consciousness levels in both resting and task states. Extremely large effect sizes validate the strength of this association, including resting-state occurrence rate ηp^2^ = 0.90, GFP ηp^2^ = 0.89, duration ηp^2^ = 0.38, and task-state HC stimulation response ηp^2^ = 0.24. Moreover, the use of a resting-state global template provided a robust and reliable analytical foundation for these findings.

Compared with prior work, our findings are broadly consistent with the view that Microstate D captures aspects of default mode network related activity and is sensitive to altered consciousness. Previous reviews and source analyses have linked Microstate D to posterior midline and parietal sources and to large scale network dynamics implicated in consciousness, which supports our anatomical and functional interpretation [22]. Recent empirical studies that applied microstate analysis in patients with prolonged Disorders of Consciousness or after brain injury likewise reported alterations in Microstate D metrics and associations with clinical level, lending external support to our resting state observations [42].

Our task-state results extend these prior reports by showing that preserved Microstate D reactivity to multisensory input distinguished MCS+ from MCS− and VS. This pattern aligns with studies indicating that posterior parietal and DMN-related dynamics relate to the capacity for stimulus driven and higher-order cognitive responses, and with multisensory EEG work showing that combined visual and olfactory inputs can engage distributed networks and modulate transient brain states [43]. Thus, the selective task reactivity we observed in MCS+ supports the idea that Microstate D dynamics reflect retained network flexibility that is necessary for more complex stimulus processing.

We addressed methodological sources of variability by constructing a resting state global template across participants, which improved comparability of microstate metrics across consciousness levels. This approach appears advantageous for capturing core, stable features in DOC patients, who showed reduced template-dependent transitions compared with healthy controls. Similar methodological concerns about template choice and intersubject variability have been raised in the microstate literature and highlight the utility of a common baseline for group comparisons [22].

However, we acknowledge that using a single resting-state template inevitably limits sensitivity to subtle task-specific reconfigurations of microstate topographies, particularly in healthy individuals who are capable of generating more differentiated task-evoked spatial patterns. In the present study, prioritizing a unified template was a deliberate methodological decision to ensure cross-state and cross-group comparability rather than to maximize detection of fine-grained task-induced spatial changes. Future work could complement this approach by constructing condition-specific or group-specific templates to directly assess whether task-related microstate reconfigurations differ across consciousness levels.

This research was subject to several limitations. The comparatively small sample size (n = 27) may elevate the risk of type II errors, meaning larger study populations are required to confirm whether the findings supported by large effect sizes can be generalized. Notably, the small sample size is partially attributable to the inherent challenges in recruiting patients with DOC. This vulnerable population is characterized by severe neurological impairment, limited accessibility to specialized care settings, and ethical considerations regarding informed consent from family members. The lack of concurrent fMRI restricted the direct verification of the spatial correspondence between Microstate D and the DMN. Additionally, the lack of longitudinal data prevented assessment of long-term intervention effects. Another limitation is that we did not perform split-half or subgroup-level clustering to formally evaluate the stability of the global microstate template. While several findings in our dataset indicate that the template is robust across groups, future studies with larger and more balanced samples should include dedicated reliability analyses. For the unresponsiveness observed in MCS− and VS patients, future studies could incorporate invasive approaches such as deep brain stimulation (DBS) to further explore the modulatory potential of neurostimulation on dynamic brain network responses. Furthermore, machine learning or deep learning-based classification of Microstate D according to disease severity, using algorithms such as Support Vector Machine (SVM), Random Forest, and Gradient Boosting Decision Tree (XGBoost), has not yet been performed. This would be valuable for further establishing its utility as a biomarker.

## 5. Conclusions

This study proposed dual-modality EEG microstate analysis method integrating resting-state and task-state data, combined with a multisensory stimulation paradigm and a resting-state global template, to systematically elucidate the dynamic brain network response mechanisms in DOC patients. The results showed that Microstate D parameters associated with the posterior parietal region followed a gradient attenuation pattern across DOC severity levels (HC > MCS+ > MCS− > VS) with very large effect sizes in the resting state. In the task state, only MCS+ patients exhibited a measurable increase in Microstate D coverage in response to combined visual and olfactory stimulation, whereas MCS− and VS patients showed no significant modulation. These findings indicated that Microstate D had the potential to serve as a specific electrophysiological biomarker for identifying the MCS+ subtype.

Clinically, Microstate D offered objective and high-temporal-resolution information that supplemented behavioral assessments, improved DOC severity evaluation and subtype differentiation, and supported the development of targeted multisensory stimulation interventions. Future studies should include larger samples, adopt longitudinal approaches, and integrate multimodal neuroimaging or neuromodulation techniques to clarify underlying neural mechanisms.

## Figures and Tables

**Table 1:** Demographic characteristics.

GROUP	HC (n = 9)	MCS+ (n = 6)	MCS− (n = 6)	VS (n = 6)	*p*
Age	28.7 ± 10.4	32.6 ± 8.4	31.5 ± 8.4	28.5 ± 8.4	>0.05
Gender (female/male)	6/3	4/2	3/3	3/3	>0.05
Mean education	12.5 ± 2.1	11.5 ± 3.1	10.6 ± 3.1	10.8 ± 2.1	>0.05
CRS-R	22.8 ± 0.1	17.8 ± 1.17	10.3 ± 1.03	6.2 ± 1.72	F = 337.76 *p* < 0.001

**Table 2:** Results of the Microstate A parameter in different masters.

Group	Template	Coverage (%)	GFP (μV)	Duration (ms)
HC	Rest	11.8 ± 3.9	4.1 ± 1.3	80.6 ± 12.2
	Task	10.2 ± 2.4	5.5 ± 0.6	78.3 ± 10.5
MCS+	Rest	12.2 ± 2.1	2.4 ± 0.6	78.6 ± 8.7
	Task	11.8 ± 1.7	4.1 ± 0.4 *	82.6 ± 5.3
MCS−	Rest	12.1 ± 1.1	3.6 ± 0.2	81.4 ± 5.3
	Task	13.7 ± 0.8	3.2 ± 0.1	82.9 ± 4.2
VS	Rest	11.1 ± 1.1	3.5 ± 0.2	82.6 ± 5.9
	Task	11.5 ± 0.5	4.6 ± 0.2	81.3 ± 5.8

Symbol * indicates *p* < 0.05.

**Table 3:** Results of the Microstate B parameter in different masters.

Group	Template	Coverage (%)	GFP (μV)	Duration (ms)
HC	Rest	12.1 ± 3.4 *	5.1 ± 1.4	90.6 ± 11.9
	Task	9.6 ± 2.9	5.7 ± 0.8	78.3 ± 10.5
MCS+	Rest	14.2 ± 1.8	4.2 ± 0.8	88.5 ± 10.7
	Task	12.9 ± 0.9	5.1 ± 0.4	83.2 ± 7.6
MCS−	Rest	13.2 ± 0.8	5.6 ± 0.3 *	87.1 ± 6.3 *
	Task	12.8 ± 0.6	4.2 ± 0.1	74.6 ± 3.9
VS	Rest	13.1 ± 0.8	3.6 ± 0.2	88.1 ± 5.9
	Task	10.2 ± 0.9	3.7 ± 0.2	79.8 ± 5.3

Symbol * indicates *p* < 0.05.

**Table 4:** Results of the Microstate C parameter in different masters.

Group	Template	Coverage (%)	GFP (μV)	Duration (ms)
HC	Rest	16.1 ± 3.2 *	7.1 ± 1.2 *	97.6 ± 10.2 *
	Task	12.1 ± 2.3	5.8 ± 0.7	82.3 ± 11.9
MCS+	Rest	14.2 ± 1.8	6.9 ± 0.6	87.5 ± 8.4
	Task	13.8 ± 1.1	5.8 ± 0.5	85.2 ± 7.3
MCS−	Rest	14.5 ± 0.5	5.2 ± 0.3	76.1 ± 6.3
	Task	13.7 ± 0.6	5.6 ± 0.1	75.2 ± 5.2
VS	Rest	11.9 ± 1.1	6.1 ± 0.3	77.2 ± 5.9
	Task	10.7 ± 0.6	4.8 ± 0.3	77.6 ± 5.6

Symbol * indicates *p* < 0.05.

**Table 5:** Results of the Microstate D parameter in different masters.

Group	Template	Coverage (%)	GFP (μV)	Duration (ms)
HC	Rest	23.5 ± 3.2 *	14.7 ± 4.1 *	115.6 ± 10.2
	Task	14.7 ± 4.1	3.8 ± 1.1	108.3 ± 12.5
MCS+	Rest	8.7 ± 1.2	4.1 ± 0.9	89.5 ± 8.7
	Task	6.6 ± 1.1	2.8 ± 0.7	85.2 ± 7.3
MCS−	Rest	5.5 ± 1.2	1.9 ± 0.6	75.1 ± 6.3
	Task	4.8 ± 0.8	0.9 ± 0.8	65.2 ± 7.2
VS	Rest	5.6 ± 0.9	1.1 ± 0.7	72.1 ± 5.9
	Task	4.9 ± 0.6	1.0 ± 0.8	63.2 ± 7.3

Symbol * indicates *p* < 0.05.

**Table 6:** Comparison of goodness-of-fit indicators.

Template	Indicator	HC	MCS+	MCS−/VS	Group-F	*p*-Value
Rest	R^2^	0.78 ± 0.06	0.52 ± 0.08	0.31 ± 0.05	28.7	<0.05
	R-S-D	2.1 ± 0.3	3.5 ± 0.5	4.8 ± 0.7	32.4	<0.05
Task	R^2^	0.55 ± 0.07	0.38 ± 0.06	0.22 ± 0.04	19.5	<0.05
	R-S-D	3.4 ± 0.4	4.7 ± 0.6	6.1 ± 0.9	25.6	<0.05

R^2^ reflects the explanatory power of the template for the data and R-S-D reflects the fitting error.

## Data Availability

The data presented in this study are available on request from the corresponding author. The data supporting the findings of this study are not publicly available due to the need to protect the privacy of human subjects, which is in line with the ethical approval requirements (Ethics Committee of Beijing Tiantan Hospital Affiliated to Capital Medical University, protocol code: KY2023-175-03).
